# Glucose-regulated protein 78 binds to and regulates the melanocortin-4 receptor

**DOI:** 10.1038/s12276-018-0144-8

**Published:** 2018-09-12

**Authors:** Ye Ran Yoon, Tae-Gul Lee, Mi-Hyun Choi, Seung Woo Shin, Young-Gyu Ko, Im Joo Rhyu, Dong-Hoon Kim, Je Kyung Seong, Ja-Hyun Baik

**Affiliations:** 1 Department of Life Sciences, Korea University, Seoul, 02841 South Korea; 2 Department of Anatomy, College of Medicine, Korea University, Seoul, 02841 South Korea; 3 Department of Medical Sciences, College of Medicine, Korea University, Seoul, 02841 South Korea; 4 Department of Pharmacology, College of Medicine, Korea University, Seoul, 02841 South Korea; 5 Laboratory of Developmental Biology and Genomics, Institute for Veterinary Science, and BK21 Program for Veterinary Science, College of Veterinary Medicine, Seoul National University, Seoul, South Korea; 6 Korea Mouse Phenotyping Center (KMPC), Seoul National University, Seoul, South Korea; 7 Interdisciplinary Program for Bioinformatics, Program for Cancer Biology, and Bio MAX Institute, Seoul National University, Seoul, South Korea

## Abstract

The melanocortin-4 receptor (MC4R) belongs to the G protein-coupled receptor (GPCR) family and plays an essential role in the control of energy homeostasis. Here, we identified a novel MC4R-interacting protein, glucose-regulated protein 78 (GRP78), from a pulldown assay using hypothalamic protein extracts and the third intracellular loop of MC4R. We found that MC4R interacted with GRP78 in both the cytosol and at the cell surface and that this interaction increased when MC4R was internalized in the presence of the agonist melanotan-II (MTII). Downregulation of GRP78 using a short interfering RNA approach attenuated MTII-mediated receptor internalization. Reduction in GRP78 expression during tunicamycin-induced endoplasmic reticulum stress also suppressed MTII-mediated internalization of MC4R and cAMP-mediated transcriptional activity. Furthermore, lentiviral-mediated short hairpin RNA knockdown of endogenous GRP78 in the paraventricular nucleus (PVN) of the hypothalamus resulted in an increase in body weight in mice fed a high-fat diet. These results suggest that GRP78 in the PVN binds to MC4R and may have a chaperone-like role in the regulation of MC4R trafficking and signaling.

## Metabolic disease: Apprehending an appetite adjuster

A molecular ‘chaperone’ helps regulate an essential metabolic signaling protein by ensuring that the protein is in the right place at the right time. The melanocortin-4 receptor (MC4R) controls food intake and energy burning. Abnormal MC4R activity is strongly linked with obesity. Little is known about how this protein is regulated, but researchers led by Ja-Hyun Baik at Korea University, Seoul, South Korea, have identified a potentially important partner for MC4R. They determined that MC4R binds a so-called ‘chaperone’ protein, GRP78, which facilitates trafficking of this receptor from the cell membrane, where it can engage in active signaling, to the cell interior. The researchers showed that GRP78 helps cells cope with cellular stress associated with metabolic disease. Absence of this protein increases sensitivity to diet-induced obesity in mice, indicating a possible target for drug development.

## Introduction

Melanocortin-4 receptor (MC4R) signaling in the brain is one of the main regulators of central energy homeostasis to control body energy balance, energy expenditure, and food intake^1–4^. Disruption of the mouse MC4R gene leads to maturity-onset obesity syndrome associated with hyperphagia, hyperinsulinemia, and hyperglycemia^5^. A conditional knockout of *Mc4r* transcription achieved by the insertion of a loxP-flanked transcriptional blocking sequence between the transcription start site and the ATG of the *Mc4r* gene also results in markedly obese mice. When such mice were bred with sim1-Cre transgenic mice, MC4R expression was restored in the paraventricular nucleus (PVN) of the hypothalamus and induced in a subpopulation of amygdala neurons. In these mice, the control of food intake was rescued, thus preventing an obese phenotype, while reduced energy expenditure was unaffected^6^, indicating a divergence in the melanocortin pathways controlling energy balance.

MC4R is a G protein-coupled seven-transmembrane domain receptor that, in the absence of a ligand, exhibits constitutive activity that results in increased basal cAMP production^7^. This constitutive activity has recently been suggested to be conferred by the tethered intramolecular ligand functionality of the N-terminal domain. Mutations in this domain that are associated with obesity appear to attenuate this activity^8^. The physiological relevance of the constitutive activity of MC4R in the context of obesity remains poorly understood. However, studies with these receptor variants suggest that the constitutive activity of MC4R is relevant to the maintenance of energy homeostasis^8^.

We have previously observed that the third intracellular loop of MC4R is essential not only for the functional activity and selectivity of G protein coupling^9,10^, but also for the regulation and maintenance of an optimal constitutive activity of MC4R^11^. The third intracellular loop of this receptor is critically required for functional conformational changes in MC4R. Furthermore, certain mutations in this region modify coupling selectivity and impair melanocortin-induced signal transduction^10,11^.

In an attempt to better understand the regulation of MC4R signaling, we sought to identify novel-binding proteins for MC4R by using its third intracellular loop as bait in hypothalamic extracts. We found that MC4R interacted with glucose-regulated protein 78 (GRP78), an endoplasmic reticulum (ER) stress-inducible molecular chaperone. We identified GRP78 expression in the PVN, where MC4R is also expressed. Under ER stress, downregulation of GRP78 using an siRNA approach significantly inhibited the internalization of MC4R and reduced the MC4R–GRP78 interaction in the hypothalamus. Furthermore, lentiviral-mediated short hairpin RNA knockdown of endogenous GRP78 in the PVN resulted in a significantly higher body weight in high-fat diet (HFD)-fed mice than in control virus-injected HFD-fed mice. Pretreatment with the pharmacological chaperone 4-phenylbutyric acid (4-PBA) inhibited the increase in food intake induced by intracerebroventricular injection of the MC4R antagonist agouti-related protein (AgRP). These data suggest that GRP78 plays an important role in the control of hypothalamic MC4R signaling, specifically in the context of energy homeostasis.

## Materials and methods

### Cell culture and ER stress induction

HEK 293T cells were treated with 2.5 µg/ml tunicamycin for 3 h in medium supplemented with 0.1% bovine serum albumin and 0.1% antibiotics to serum-starve the cells (see Supplementary Materials and Methods for details).

### Mice

Ten–twelve-week-old male C57BL/6J *ob/ob* mice and wild-type (WT) mice (Jackson Laboratories, Bar Harbor, ME) were used. All animals were housed under a 12-h light/dark cycle under constant conditions of temperature and humidity and had access to tap water and regular diet ad libitum (see Supplementary Materials and Methods for details).

### Plasmid construction for the GRP78 domain

The sequence of the Chinese hamster GRP78 cDNA was obtained from the NCBI database (M17169). GlobPlot algorithm^12^ was used to design the following GRP78 functional domains (see Supplementary Materials and Methods for details).

### Statistical analysis

See Supplementary Materials and Methods for statistical analysis.

### Other methods

Additional experimental procedures are provided in the Supplementary Materials and Methods.

## Results

### Identification of GRP78 as a novel-binding protein of the third intracellular loop of MC4R

**Fig. 1:**
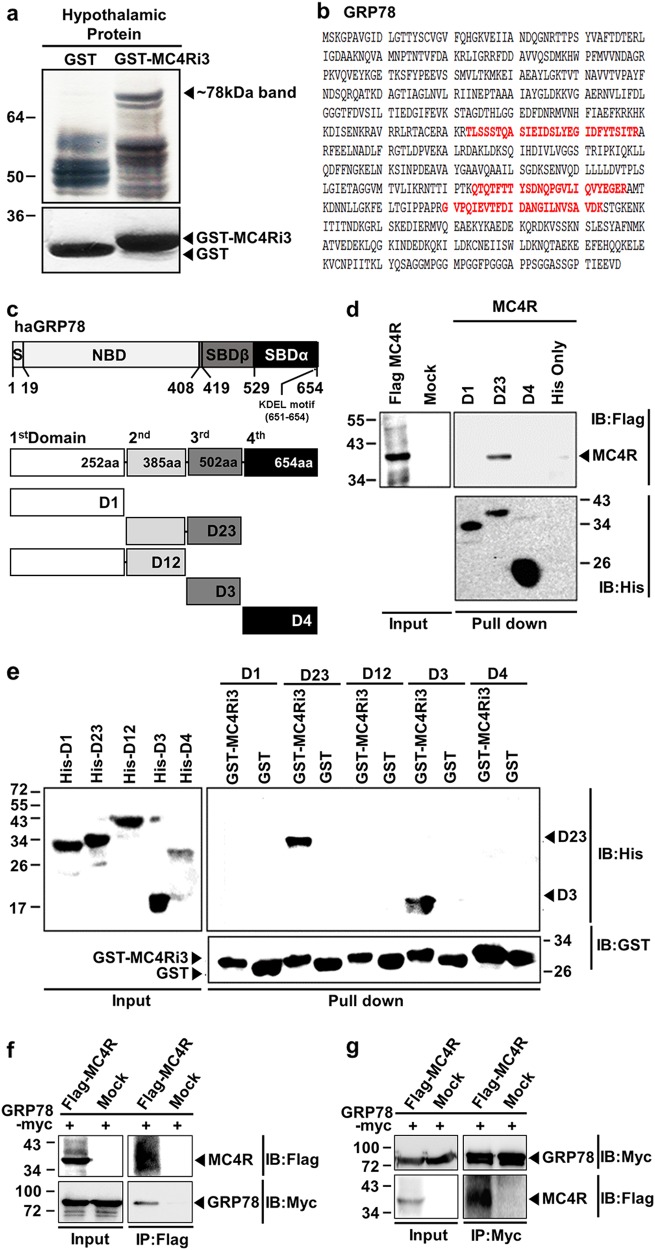
Identification of GRP78 as a binding protein for the third intracellular loop of MC4R in the hypothalamus. Bacterially expressed GST alone and GST-MC4Ri3 proteins were incubated with mouse hypothalamic extracts. Pulldown complexes were separated by SDS-PAGE and visualized by silver staining (**a**, upper gel). Coomassie blue staining shows immobilized GST alone or GST-MC4Ri3 proteins (**a,** lower gel). An ~78-kDa band specific to the GST-MC4Ri3 pulldown sample was isolated and sequenced by MALDI-TOF MS/MS analysis. **b** Amino acid (a.a.) sequence of GRP78. Peptide sequences obtained by MALDI-TOF MS/MS are indicated in red. **c** Schematic diagram of globular domains within Chinese hamster GRP78 (haGRP78); signal sequence (S: 1–18), nucleotide-binding domain (NBD: 19–407), substrate-binding domains (SBDβ, 419–528 and SBDα, 529–654) with an ER retention signal KDEL motif (aa 651–654) and the five globular domain fragments (a.a. 1–252, 253–502, 1–385, 386–502, 503–654). Domain fragments are represented as D1, D23, D12, D3, and D4, respectively. **d** His-tag pulldown of bacterially expressed and purified GRP78 domain fragments with 3xFlag-MC4R expressed in HEK 293T cells. MC4R was incubated with His-tagged proteins coupled to Ni-NTA agarose beads. **e** GST pulldown assay showing the interaction between GST-MC4Ri3 and the third globular domain of GRP78. To confirm the MC4R–GRP78 interaction in vitro, coimmunoprecipitation was performed using membrane proteins obtained from HEK 293T cells transfected with 3xFlag-MC4R or GRP78-Myc plasmids. **f** Membrane proteins were immunoprecipitated with anti-Flag and then immunoblotted with anti-Myc. **g** Membrane proteins were immunoprecipitated with anti-Myc and then immunoblotted with anti-Flag

To identify potential-binding proteins of the third intracellular loop of MC4R, we purified GST-fusion proteins encompassing this loop from bacteria, conjugated them to glutathione Sepharose 4B beads, and used them as bait to perform GST-tag pulldown assays in mouse hypothalamic extracts. As shown in Fig. 1a, we identified a specific band at ~70 kDa that was not present in the GST-mock vector pulldown control. We eluted at a larger scale to further visualize silver staining. This 70-kDa band was cut out and subjected to in-gel trypsin digestion and matrix-assisted laser desorption ionization time-of-flight mass spectrometry; analysis identified the peptides as glucose-regulated protein of 78 kDa (GRP78) (Fig. 1b). We performed this proteomic analysis 12 times, and in most of the experiments, GRP78 was identified as a candidate-binding protein.

GRP78 contains an ER signal peptide (aa 1–18) at the N terminus, a nucleotide-binding domain (19–407 amino acid residue), substrate-binding subdomain α (529–654), substrate-binding subdomain β(419–528), and an ER retention signal KDEL motif (aa 651–654) at the C terminus^13–18^. The GlobPlot algorithm^12^ partitioned GRP78 into four functional domains, with the first domain (D1) containing amino acid residues 1–252, second domain (D2) containing residues 253–385, third domain (D3) containing residues 386–502, and fourth domain (D4) containing residues 503–654. D1 and D2 included the ATPase domain, D3 included the peptide-binding domain, and D4 included the C-terminal tail. Based on this analysis, we subcloned five constructs, each encoding a GRP78 domain fragment with a polyhistidine tag on its C-terminal (Fig. 1c and Supplementary Table 1) tail and named them D1, D23, D12, D3, and D4. To identify the interaction between MC4R and each of the GRP78 fragments, we performed pulldown assays using lysates from 3xFlag-MC4R-expressing HEK 293T cells and bacterially expressed and purified His-tagged GRP78 domain fragments (D1, D23, and D4). Analysis of bound proteins by western blot using the FLAG antibody revealed a direct interaction between D23 and MC4R (Fig. 1d). Furthermore, we performed pulldown assays using GST-tagged MC4Ri3 and D1, D23, D12, D3, and D4. The results in Fig. 1e show that D23 and D3 were bound to MC4Ri3, indicating that the D3 domain, which contains the peptide-binding domain of GRP78, interacted with MC4Ri3.

To examine whether GRP78 can bind to MC4R in mammalian cells, plasmids coding GRP78-Myc and 3xFlag-MC4R were cotransfected in HEK 293T cells, and the cellular membranes were isolated. A coimmunoprecipitation assay was performed using the membrane fraction and Myc antibody, followed by western blot analysis using the Flag antibody, and vice versa. We found that GRP78 and MC4R indeed interacted with each other at the cellular membrane (Fig. 1f, g).

### Cellular colocalization of MC4R and GRP78

**Fig. 2:**
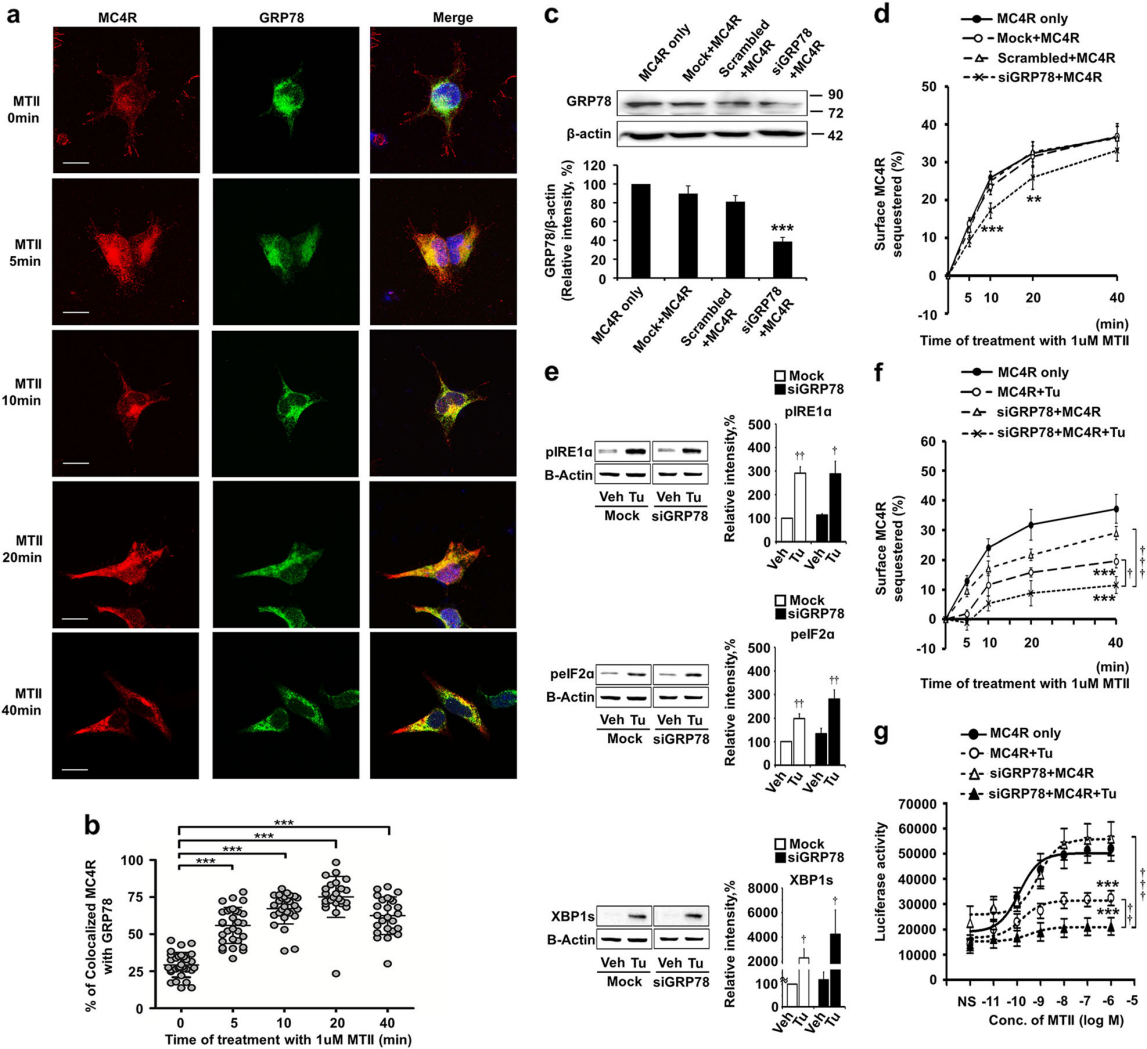
Cellular colocalization of MC4R and GRP78, and GRP78 downregulation of MC4R trafficking and intracellular signaling. The intracellular localization of MC4R and GRP78 was analyzed in the absence and presence of the MC4R agonist MTII in HEK 293T cells overexpressing 3xFlag-MC4R and GRP78-Myc plasmids. To observe the effect of GRP78 knockdown, HEK 293T cells were sequentially transfected with siRNA against GRP78 (siGRP78) and the 3xFlag-MC4R plasmid. siGRP78 and 3xFlag-MC4R-expressing HEK 293T cells were treated with 2.5 µg/ml tunicamycin (Tu) to induce ER stress. Surface receptors were measured by ELISA, and intracellular signaling was analyzed by a CRE-mediated luciferase assay. **a** Time course of MTII treatment of HEK 293T cells expressing 3xFlag-MC4R (labeled with Alexa Fluor 568, red) and GRP78-Myc (labeled with Alexa Fluor 488, green) proteins. After treatment with 1 µM MTII, cells were fixed at 5, 10, 20, and 40 min and immunostained (Scale bar, 20 µm). **b** Quantification of colocalized MC4R with GRP78. Student’s *t* test for **b**: 0 min, *n* = 31; 5 min, *n* = 30; 10 min, *n* = 28; 20 min, *n* = 24; 40 min, *n* = 25; ***P < 0.0001 versus 0 min control. **c** Downregulation of GRP78 in siGRP78-transfected cells compared to that in control-transfected cells. Control plasmids included a mock (empty vector lacking a siRNA sequence) and a scramble (vector containing an siRNA sequence that was designed not to degrade any specific cellular message). **d** The percentage of sequestered MC4R, shown as a ratio of the total number of receptors to internalized receptors, after GRP78 knockdown. **e** Expression of ER stress marker proteins after ER stress induction: phosphorylated inositol-requiring enzyme 1α (pIRE1α), phosphorylated eukaryotic initiation factor 2α (peIF2α), and spliced X-box-binding protein 1 (XBP1s). After treatment with 2.5 µg/ml tunicamycin for 3 h in cells transfected with control mock vector or with siGRP78, cell lysates were analyzed for levels of pIRE1α, peIF2α, XBP1s, and β-actin (as a control for normalization) by western blotting. **f** The ratio of the total receptors to internalized receptors under GRP78 knockdown and ER stress to illustrate the percentage of sequestered MC4Rs. **g** Relative cAMP-mediated transcriptional activity stimulated with various concentrations of MTII during GRP78 knockdown and ER stress. The results are shown as the mean ± SEM of at least three independent experiments performed in triplicate. One-way ANOVA, Bonferroni post hoc tests for **c**; two-way ANOVA, Bonferroni post hoc tests for **d**, **f**, and **g**: *P, ^†^*P* < 0.05, **P, ^††^*P* < 0.001, and ***P, ^†††^*P* < 0.001; *, *P*, versus MC4R only or si-mock; †, *P*, versus siGRP78 + MC4R + Tu or vehicle; One-tailed Student’s *t* test for **e**: ^†^*P* < 0.05, ^††^*P* < 0.001, and ***P < 0.001; *, *P*, versus si-mock; †, *P*, versus vehicle. The data are presented as the mean ± SEM

Next, we analyzed the intracellular localization of MC4R and GRP78 in the absence and presence of the MC4R agonist MTII in HEK 293T cells overexpressing 3xFlag-MC4R and GRP78-Myc plasmids (Fig. 2a). Confocal microscopy revealed the partial colocalization of MC4R (red) with GRP78 (green) in the non-MTII-treated control (29.19 ± 1.48%) (Fig. 2b). However, in the presence of MTII, colocalization of MC4R with GRP78 increased as MC4R internalization increased with agonist incubation time (5 min: 55.85 ± 2.22%, 10 min: 67.22 ± 1.95%, 20 min: 75.15 ± 2.82%, and 40 min: 62.48 ± 2.53% versus 0 min: 29.19 ± 1.48%, *n* = 24–31, two-tailed Student’s *t* test, *P* < 0.0001) (Fig. 2b). These results indicate that MC4R and GRP78 interact in both the cytosol and at the cell surface and that this interaction was increased as MC4R was internalized in the presence of an agonist.

### Effects of GRP78 downregulation on MC4R trafficking and intracellular signaling under ER stress induction

Because our data revealed that GRP78 could bind to MC4Ri3, we hypothesized that this interaction plays a role in regulating the G protein-mediated downstream signaling of MC4R by affecting the rate of receptor internalization. We thus examined whether knockdown of GRP78 could affect the internalization of MC4R. The cells were transiently transfected with the 3xFlag-MC4R plasmid along with the control mock vector, control scrambled siRNA, or siGRP78. SiGRP78 was transfected into HEK 293T cells to reduce the level of GRP78 expression. We confirmed that GRP78 expression was markedly reduced at the protein level in siGRP78-transfected cells but not in the mock vector- or scrambled siRNA-transfected cells (Fig. 2c). Changes in cell surface receptors after incubation with 1 µM MTII for different time intervals (0, 5, 10, 20, and 40 min) were measured by ELISA using an anti-Flag antibody (Fig. 2d). Treatment with MTII resulted in a time-dependent sequestration of MC4R; rapid MC4R internalization was observed from the beginning of treatment and within 40 min, and 40% of the receptors initially present on the cell surface were internalized (Fig. 2d). However, knockdown of GRP78 with siRNA attenuated the MTII-mediated receptor internalization during this period (the percentage of internalized receptors was as follows for MC4R transfection versus siGRP78 and MC4R cotransfection: 5 min: 13.74 ± 1.11% versus 9.19 ± 1.39%; 10 min: 25.90 ± 1.64% versus 17.21 ± 2.04%; 20 min: 32.32 ± 3.20% versus 25.96 ± 3.12%; 40 min: 36.61 ± 3.03% versus 33.08 ± 2.70%; *n* = 6, two-way repeated measures (RM ANOVA), Bonferroni post hoc tests, *P* < 0.01 and *P* < 0.001) (Fig. 2d). These results suggest that GRP78 was involved in the MTII-mediated internalization of MC4R.

ER stress is now well accepted to be involved in the pathology of various diseases, including metabolic disorders^19,20^. Given that GRP78 is a key player in ER stress, we examined how GRP78 knockdown can affect MC4R trafficking under ER stress conditions. To pharmacologically induce ER stress, cells were treated with tunicamycin, along with 1 µM of MTII for the times indicated. ER stress induced by tunicamycin treatment was confirmed by monitoring the expression of several ER stress markers, such as phosphorylated inositol-requiring enzyme 1α (pIRE1α), which is a downstream target of the protein kinase RNA-like endoplasmic reticulum kinase (PERK) pathway, phosphorylated eukaryotic initiation factor 2α (peIF2α), and spliced X-box-binding protein 1 (XBP1s). Western blot analysis revealed that induction of ER stress by tunicamycin resulted in an increase in pIRE1α, peIF2α, and XBP1s expression (pIRE1α: 290.85 ± 26.87% of vehicle group, *n* = 5, one-tailed Student’s *t* test, *P* = 0.001; peIF2α: 197.64 ± 20.52% of vehicle group, *n* = 7, one-tailed Student’s *t* test, *P* = 0.0016; XBP1s: 2307.22 ± 718.89% of vehicle group, *n* = 7, one-tailed Student’s *t* test, *P* = 0.0110, Fig. 2e). In addition, we observed that the expression levels of these ER stress marker proteins were also increased with tunicamycin treatment under GRP78 knockdown conditions (siGRP78 + vehicle versus siGRP78 + tunicamycin; pIRE1α: 113.04 ± 5.55% versus 287.51 ± 53.50%, *n* = 5, one-tailed Student’s *t* test, *P* = 0.0196; peIF2α: 134.02 ± 22.75% versus 280.97 ± 38.96%, *n* = 7, one-tailed Student’s *t* test, *P* = 0.0049; XBP1s: 119.61 ± 33.15% versus 4233.36 ± 1966.88%, *n* = 7, one-tailed Student’s *t* test, *P* = 0.0390, Fig. 2e). These data indicate that GRP78 knockdown enhances UPR activation upon ER stress.

Changes in the number of MC4Rs at the cell surface were measured by ELISA using an anti-Flag antibody (Fig. 2f). We found that MTII-mediated internalization of MC4R was markedly suppressed by tunicamycin treatment. SiRNA knockdown of GRP78 during tunicamycin treatment further aggravated the attenuation of MC4R internalization (siGRP78 + MC4R versus siGRP78 + MC4R + tunicamycin, at 5 min: 9.63 ± 1.88% versus −1.13 ± 1.85%; at 10 min: 17.17 ± 0.95% versus 5.38 ± 0.98%; at 20 min: 21.70 ± 1.03% versus 8.79 ± 3.97; at 40 min: 29.01 ± 0.95% versus 11.54 ± 0.68%; MC4R + tunicamycin versus siGRP78 + MC4R + tunicamycin, at 5 min: 1.88 ± 1.37% versus −1.13 ± 1.85%; at 10 min: 11.61 ± 3.59% versus 5.38 ± 0.98%; at 20 min: 15.86 ± 1.57% versus 8.79 ± 3.97; at 40 min: 19.60 ± 2.21% versus 11.54 ± 0.68%; *n* = 5, two-way RM ANOVA, Bonferroni post hoc tests; at 40 min, siGRP78 + MC4R + tunicamycin compared to siGRP78 + MC4R or MC4R + tunicamycin, *P* < 0.001 and *P* < 0.05, respectively) (Fig. 2f).

We next examined the combined effect of GRP78 knockdown and ER stress on the alteration of MC4R signaling. We measured the induction of CRE-luciferase reporter gene activity upon stimulation with MTII (Fig. 2g). MC4R-induced CRE-mediated transcriptional activity was notably decreased in siGRP78-transfected cells under ER stress induction (siGRP78 versus siGRP78 + ER stress: 55951.47 ± 6612.23 (EC50, 7.89E−10) versus 21141.80 ± 3466.00 (EC50, 3.43E−10); ER stress versus siGRP78 + ER stress: 32352.73 ± 2920.34 (EC50, 1.57E−10) versus 21141.80 ± 3466.00 (EC50, 3.43E−10); *n* = 5, two-way RM ANOVA, Bonferroni post hoc tests, *P* < 0.001 and *P* < 0.01, respectively) (Fig. 2g).

### Expression of GRP78 in the PVN of *ob/ob* mice

**Fig. 3:**
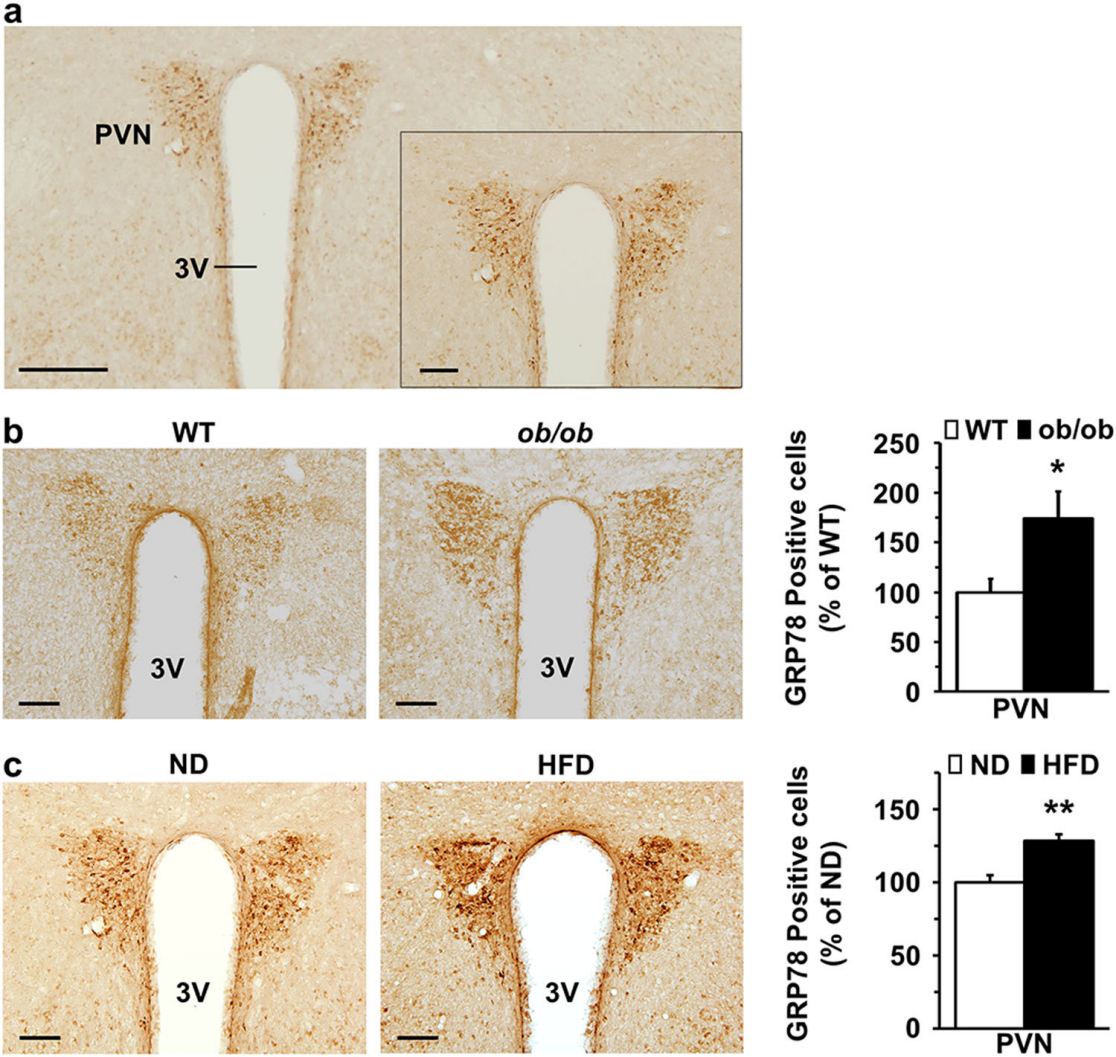
GRP78 expression in the PVN of *ob/ob* and diet-induced obese mice. Sections were prepared from the obese mouse models: 10–12-week-old *ob/ob* male mice and high-fat diet (HFD)-fed DIO mice. WT and normal diet (ND)-fed male mice were used as controls. Representative coronal sections (40 µm) stained with an antibody against GRP78 and visualized by 3,3ʹ-diaminobenzidine reactions are shown. **a** GRP78 expression in the hypothalamic PVN region. The inset in the panel shows the high-magnification area of the PVN within the low-magnification image (scale bar: low: 250 µm, high: 100 µm). **b** Representative images of GRP78 staining of sections from the PVN of *ob/ob* and WT mice (scale bar, 100 µm). **c** Representative GRP78 staining images in the PVN region of DIO mice (HFD) and control mice (ND) (scale bar, 100 µm). The number of GRP78-positive cells in the PVN was counted using stereological analysis, and the graph shows the percentage of the average total number of neurons/nucleus/WT or ND mice. One-tailed Student’s *t* test for **b**: *n* = 4 per group, *P < 0.05 versus WT mice; One-tailed Student’s *t* test for **c**: *n* = 4–5 per group, **P < 0.01 versus ND-fed mice. The data are presented as the mean ± SEM

We next explored the expression of GRP78 in the hypothalamus and observed high expression of GRP78 in the PVN, where MC4R is also highly expressed (Fig. 3a). We examined the expression level of GRP78 in the PVN of a leptin-deficient obese mouse model, *ob/ob* mice. Immunostaining and stereological analysis of coronal brain sections corresponding to the PVN prepared from *ob/ob* and WT mice revealed that the number of GRP78-positive cells was significantly greater in the PVN region of *ob/ob* mice than in that of the WT mice (ob/ob versus WT, 174 ± 27%, *n* = 4, one-tailed Student’s *t* test, *P* = 0.0250, Fig. 3b).

We also examined the expression of GRP78 in the PVN region of diet-induced obese (DIO) mice as another obese mouse model. Ten-week-old male mice were randomly divided into a control group (fed a normal diet (ND)) and a HFD group (a group fed a high-fat diet (HFD)). After 13 weeks of HFD feeding, the mice in the HFD group with obesity manifested a body weight greater than 20% of the mean value of the body weight of controls, with an average body weight of 36.07 ± 1.30 g compared to 26.40 ± 0.49 g in the ND group. We performed immunostaining and stereological analysis for GRP78-positive neurons in the PVN region of ND- and HFD-fed mice. Significantly, more GRP78-positive cells were found in the PVN region of HFD-fed mice than in that of ND-fed mice (HFD: 128.48 ± 4.35% versus ND control, *n* = 4–5, one-tailed Student’s *t* test, *P* = 0.0017, Fig. 3c).

### Regulation of GRP78 and MC4R in the hypothalamus under ER stress

**Fig. 4:**
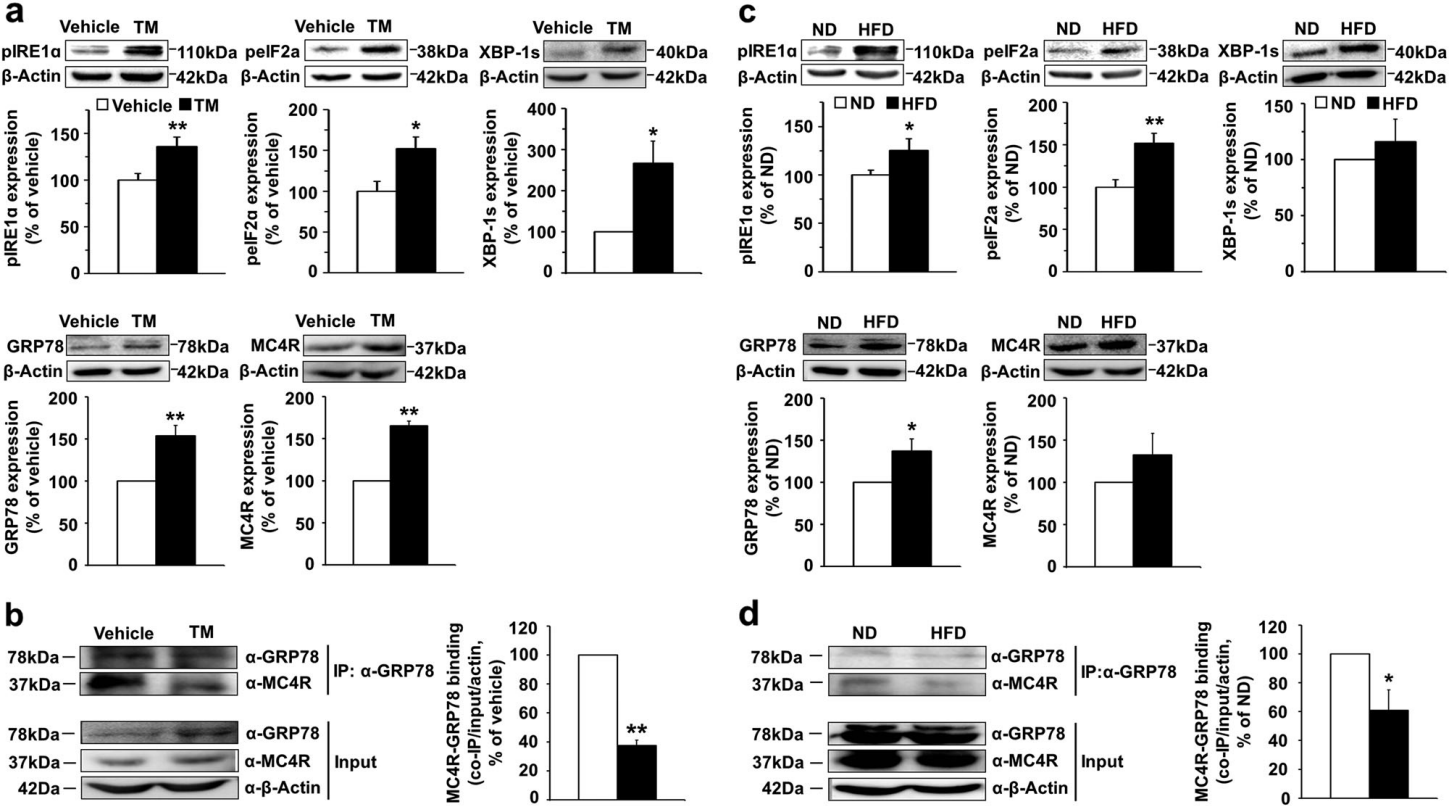
Regulation of GRP78 and MC4R in the hypothalamus under ER stress. Mice were exposed to an ER stressor, and their brains were removed. Hypothalamic protein extracts were subjected to western blotting and coimmunoprecipitation. For acute ER stress conditions, 10-week-old male mice were injected with tunicamycin (TM) or vehicle (DMSO) into the lateral ventricle (i.c.v., TM 80 µg/3.2 µl, twice every 6 h) and were killed 12 h after the first injection. For chronic ER stress conditions, 10–14-week-old male mice were fed a high-fat diet (HFD, 58.0% fat) or a normal diet (ND) for 20 weeks. The expression levels of ER stress marker proteins (pIRE1α, peIF2α, and XBP1s), GRP78, and MC4R in mice subjected to **a** acute ER stress (vehicle DMSO versus TM) or **c** chronic ER stress (ND versus HFD) were measured using western blotting. The interaction between GRP78 and MC4R was measured using coimmunoprecipitation under **b** acute ER stress or **d** chronic ER stress conditions. One-tailed Student’s *t* test for **a**–**d**: TM, *n* = 3–9 per group; HFD, *n* = 5–7 per group, *P < 0.05, **P < 0.01 versus vehicle DMSO or ND group. The data are presented as the mean ± SEM

GRP78 and MC4R had similar expression patterns in the brain; we therefore asked whether GRP78 regulated the in vivo function of MC4R, specifically under ER stress. For these experiments, a cannula was implanted into the lateral ventricle for two intracerebroventricular (i.c.v.) deliveries (6 h apart) of tunicamycin (80 µg/3.2 µl), a well-known ER stress inducer, or vehicle. Mice were killed at 12 h after the first injection. Hypothalamic lysates obtained from mice treated with tunicamycin were subjected to western blotting or coimmunoprecipitation, and the expression levels of ER stress marker proteins and GRP78 were examined. Immunoblots showed that the induction of acute ER stress by tunicamycin treatment resulted in higher levels of pIRE1α, peIF2, and XBP1s than vehicle treatment (pIRE1α: 135.79 ± 10.01% of vehicle group, *n* = 8–9, one-tailed Student’s *t* test, *P* = 0.006; peIF2α: 152.05 ± 14.46% of vehicle group, *n* = 6, one-tailed Student’s *t* test, *P* = 0.0103; XBP1s: 266.47 ± 53.93% of vehicle group, *n* = 4, one-tailed Student’s *t* test, *P* = 0.0269, Fig. 4a). Increasing levels of GRP78 and MC4R proteins were detected in the hypothalamic extracts of mice treated with tunicamycin compared to those of mice treated with vehicle (GRP78: 153.85 ± 12.35% over the vehicle group, *n* = 5, one-tailed Student’s *t* test, *P* = 0.0060; MC4R: 165.41 ± 4.79% over the vehicle group, *n* = 3, one-tailed Student’s *t* test, *P* = 0.0027, Fig. 4a). We also examined the interaction between GRP78 and MC4R in hypothalamic extracts from tunicamycin-injected mice. Although the expression levels of GRP78 and MC4R were increased by tunicamycin-induced ER stress, the interaction between these two proteins was significantly decreased (37.58 ± 3.59% of vehicle group, *n* = 3, one-tailed Student’s *t* test, *P* = 0.0016) (Fig. 4b).

ER stress and activation of the unfolded protein response (UPR) have been shown to be increased in the hypothalamus of obese mice^21^. To examine the regulation of GRP78 and MC4R under chronic ER stress conditions, 10–14-week-old male C57BL/6J mice were fed a normal diet (ND) or HFD for a period of 20 weeks, and then, the expression levels of XBP1 and GRP78 were examined in the hypothalamic extracts from these mice. As shown in Fig. 4c, higher levels of pIRE1α, peIF2α, and XBP1s proteins were detected in the hypothalamic extracts of HFD-fed mice than in those of ND-fed mice (pIRE1α: 125.14 ± 12.35% of ND group, *n* = 7, one-tailed Student’s *t* test, *P* = 0.0414; peIF2α: 151.81 ± 11.43% of ND group, *n* = 7, one-tailed Student’s *t* test, *P* = 0.0018; XBP1s: 115.78 ± 20.27% of ND group, *n* = 5, one-tailed Student’s *t* test, *P* = 0.2399), indicating that HFD caused hypothalamic ER stress. The expression levels of GRP78 and MC4R were higher in the HFD-fed mice than in the ND-fed mice, but this difference in MC4R expression did not achieve statistical significance (GRP78, 137.0 ± 14.90% of ND group, *n* = 5, one-tailed Student’s *t* test, *P* = 0.0340, MC4R, 132.52 ± 25.34% of ND group, *n* = 6, one-tailed Student’s *t* test, *P* = 0.1279) (Fig. 4c). In addition, the interaction between MC4R and GRP78 was attenuated in the hypothalamic extracts from HFD-fed mice compared to that in the extracts from ND-fed mice (60.83 ± 14.24% of ND group, *n* = 6, one-tailed Student’s *t* test, *P* = 0.0201) (Fig. 4d). These data suggest that under ER stress, although the protein level of GRP78 was increased due to the decreased interaction between MC4R and GRP78, MC4R trafficking and signaling were perturbed.

### Effects of shRNA lentivirus-mediated GRP78 knockdown in the PVN

**Fig. 5:**
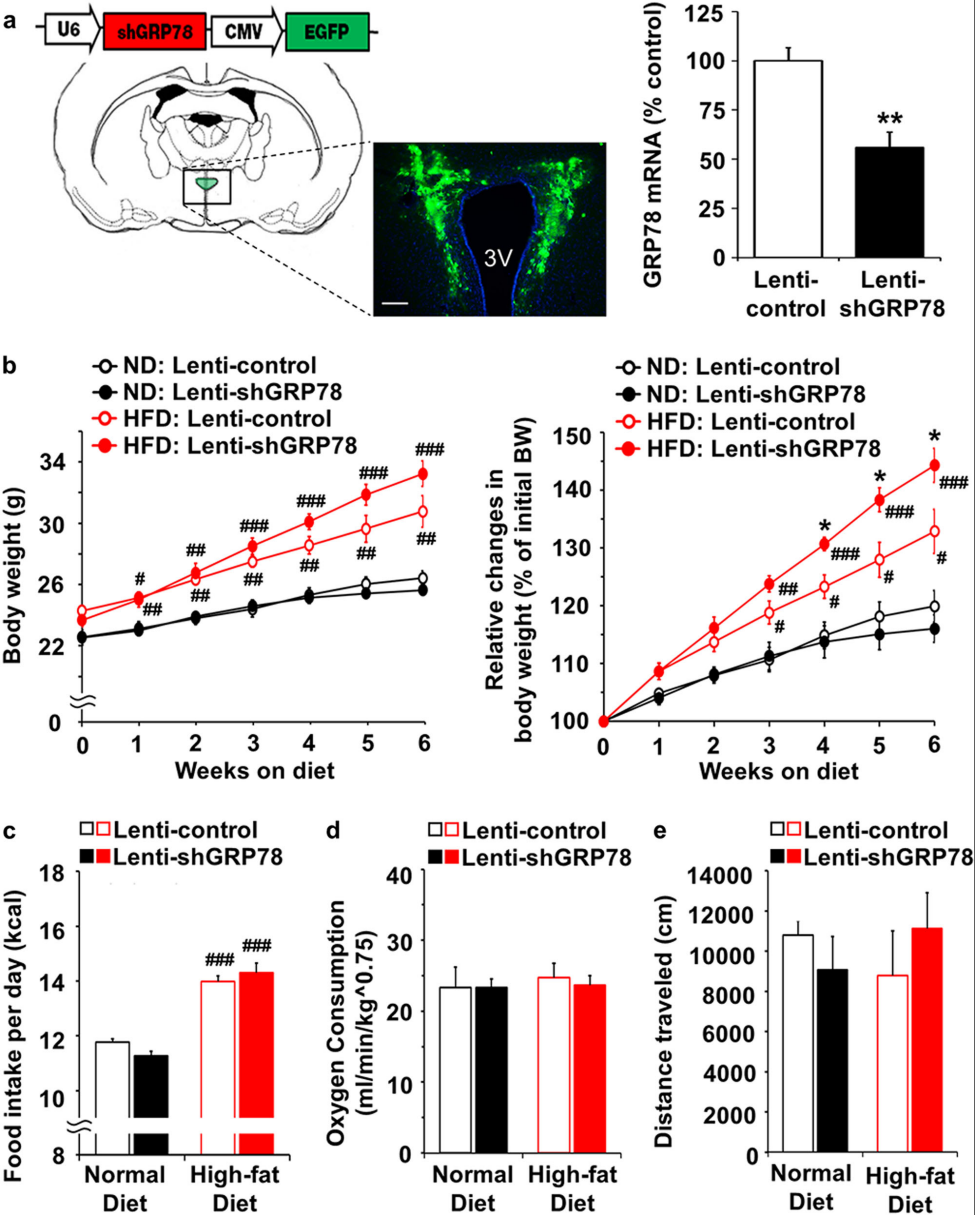
Physiological effects of lentivirus-mediated GRP78 knockdown in the PVN. Lenti-shGRP78 or Lenti-control viruses were stereotaxically delivered to the PVN of 6-week-old male mice. After surgery and recovery (for 7 days), mice were fed an ND or HFD for 40 days. Body weight and food intake were measured daily at 5:00 p.m. Before killing, oxygen consumption (for 24 h) and locomotor activity (for 1 h) were measured. **a** Schematic of the lentiviral vector-encoding GRP78 short hairpin RNA (top), and the injection sites in the PVN shown in a coronal diagram of the brain (bottom) (scale bar, 100 µm). Real-time PCR analysis of GRP78 mRNA in the PVN. Changes in **b** body weight, **c** food intake, **d** oxygen consumption, and **e** locomotor activity in mice fed an ND or HFD. One-tailed Student’s *t* test for **a**: *n* = 3 per group, **P < 0.01 versus Lenti-control. Two-way ANOVA, Bonferroni post hoc tests for **b**–**e**: *n* = 3–4, ^#^*P* < 0.05, ^##^*P* < 0.01, ^###^*P* < 0.001 versus ND, *P < 0.01 versus Lenti-control. The data are presented as the mean ± SEM

To further assess the role of GRP78 in the MC4R-mediated regulation of energy homeostasis, we constructed a lentiviral vector (Lenti-shGRP78) to reduce GRP78 expression that was delivered to the PVN bilaterally (Fig. 5a). Specific attenuation of GRP78 expression in the PVN was confirmed by real-time RT-PCR analysis of PVN tissue from injected mice compared to that of PVN tissue from control lentivirus (Lenti-control)-injected mice (55.89 ± 7.78% of Lenti-control, *n* = 3, one-tailed Student’s *t* test, *P* = 0.0063) (Fig. 5a). Six-week-old mice were injected with Lenti-control or Lenti-shGRP78, and 1 week after the injection, the mice were divided into two groups: one group received an ND, while the other group received an HFD for 40 days. Among the HFD-fed mice, knockdown of GRP78 in the PVN resulted in a significant increase in body weight compared to Lenti-control injection (both unilaterally and bilaterally, *n* = 3–4, two-way ANOVA Bonferroni post hoc tests; 5-week Lenti-control, 127.95 ± 3.00% versus Lenti-shGRP78, 138.33 ± 2.04%, shGRP78 × HFD interaction F_1,11_ = 6.61, *P* = 0.0260; 6-week Lenti-control, 132.87 ± 3.81% versus Lenti-shGRP78, 144.29 ± 2.91%, shGRP78 × HFD interaction F_1,11_ = 5.92, *P* = 0.0333) (Fig. 5b). Food intake was increased in the Lenti-shGRP78 group but not significantly. In the ND-fed mice, knockdown of GRP78 did not affect body weight or food intake compared to those in the Lenti-control group (Fig. 5c). Finally, there were no differences in oxygen consumption or locomotor activity between the Lenti-control and Lenti-shGRP78 groups (Fig. 5d, e).

### Effect of the chemical chaperone 4-PBA on the MC4R-mediated regulation of body weight and food intake

**Fig. 6:**
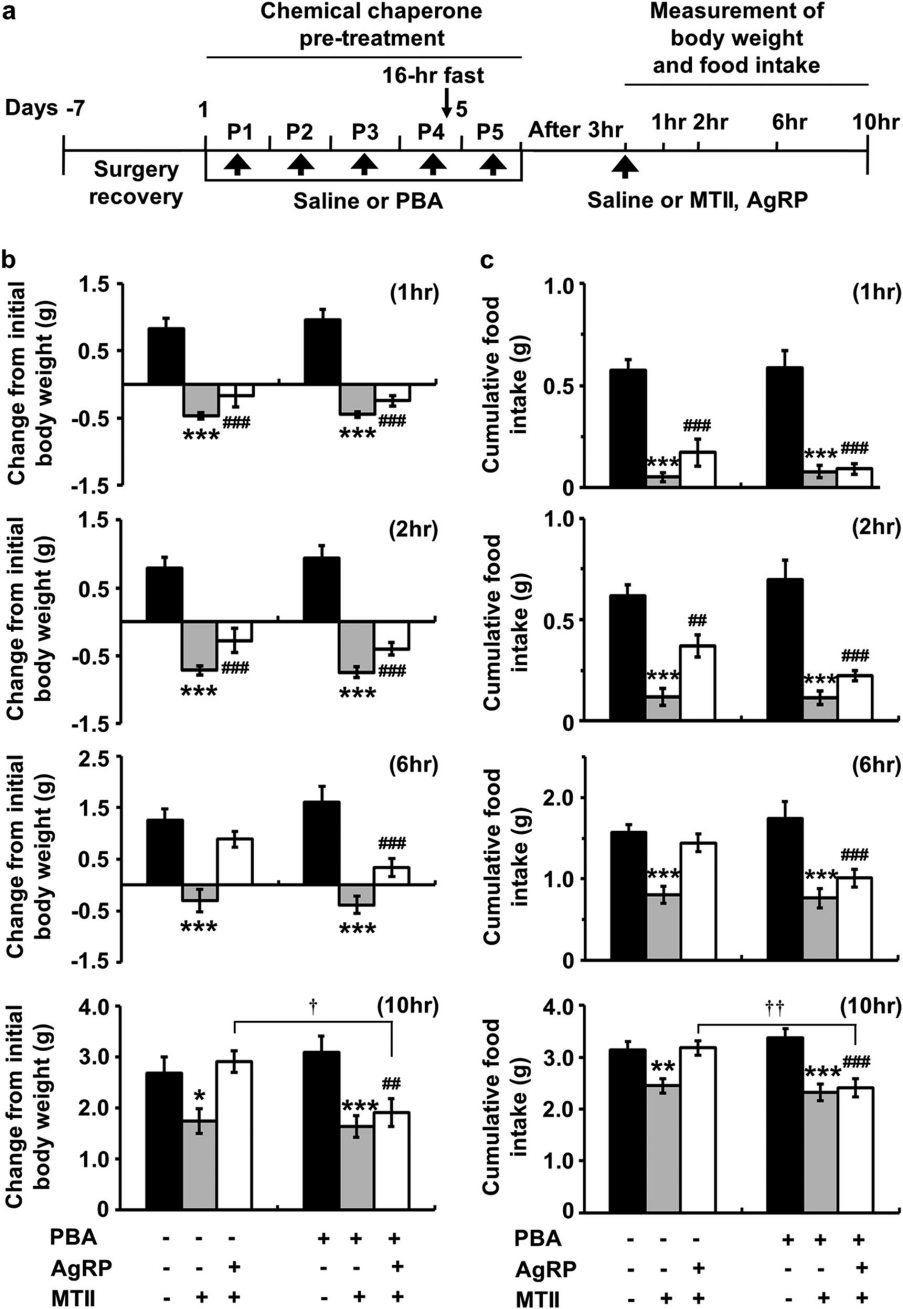
Effect of the chemical chaperone 4-PBA on the MC4R-mediated regulation of body weight and food intake. **a** Experimental protocol: 10-week-old male mice were injected with saline or 4-PBA (200 mg/kg, ip) for 5 days; on the 5th day, mice fasted for 16 h were treated with AgRP (2 nmol, i.c.v.) or MTII (3 nmol, i.c.v.). At different time points after each injection, the changes in **b** body weight and **c** food intake were measured. Two-way ANOVA, Bonferroni post hoc tests for **b**–**c**: *n* = 9 per group, *P < 0.05, **P < 0.01, ***P < 0.001, vehicle versus MTII; ^##^*P* < 0.01, ^###^*P* < 0.001, vehicle versus AgRP + MTII; ^†^*P* < 0.05, ^††^*P* < 0.05, non-4-PBA-treated versus 4-PBA-treated group. The data are presented as the mean ± SEM

Given that GRP78 appeared to be able to regulate MC4R function in vivo, we next asked whether this interaction was associated with the role of GRP78 as a molecular chaperone. To test this, we assessed the effect of a chemical chaperone, 4-PBA, on the MC4R-mediated control of body weight and food intake. Cannulas were implanted into the lateral ventricles for an i.c.v. delivery in 8–9-week-old male mice; these mice were allowed to recover for 7 days following surgery. After pretreatment with 4-PBA or saline (control) for 5 days, the mice were administered either 3 nmol MTII or 2 nmol AgRP. We measured the body weight and food intake at different time points beginning when MTII or AgRP was first delivered (Fig. 6a). At 1 and 2 h, there were no differences between the 4-PBA-pretreated and saline control groups; both groups showed a decrease in body weight and food intake upon MTII administration and exhibited a similar but smaller effect after MTII + AgRP administration. By 6 h, the body weight and food intake of the saline and AgRP-injected group recovered, while in the 4-PBA-treated group, the MTII-induced decrease in body weight and food intake was maintained even with AgRP treatment (Fig. 6b, c). At 10 h after injection of MTII or AgRP, the MTII-induced decrease in body weight and food intake in the 4-PBA-pretreated group was still maintained. Even with AgRP treatment, there was a significant difference between the saline control and 4-PBA-pretreated groups (Fig. 6b, c). Indeed, in AgRP-treated animals of the saline control group, body weight and food intake were completely recovered in the nontreated control compared to those in the MTII-injected animals. However, in the 4-PBA-treated group, the effect of MTII on body weight and food intake was significantly preserved during the same period (Fig. 6b, c and Supplementary Table 2). These results indicate that chemical chaperones, such as 4-PBA, can reduce the antagonistic effect of AgRP on body weight and food intake and further support our hypothesis that chaperone proteins play a critical role in regulating the physiological functions of MC4R in energy balance.

## Discussion

Despite recent findings that uncovered the role of MC4R in brain circuits implicated in energy homeostasis in animal models and humans, the association between MC4R signaling and its physiological function remains to be clarified. In the present study, we identified an interacting protein of MC4R, GRP78, which appears to play an important role in MC4R trafficking, particularly under cellular stress.

Considerable evidence has indicated that variable cellular stress can induce ER stress, leading to activation of the UPR, which coordinates adaptive and apoptotic responses in reaction to cellular stress^21–23^. Perturbations of this regulation are linked to multiple metabolic disorders, such as obesity and type 2 diabetes^19,21–23^. GRP78 is known as a central regulator of ER stress signaling and an important modulator of UPR signaling^24^ and has been considered a potential therapeutic target to manage ER stress-associated metabolic disorders. In this context, our identification of the interaction between GRP78 and MC4R provides a potential key signaling network to target as a treatment for hypothalamic ER stress and obesity. In the present study, we used protein pulldown assays, immunoprecipitation, and western blot techniques to demonstrate that GRP78 binds to MC4R. Although GRP78 is known primarily as an ER-associated protein^25^, recent studies have shown that GRP78 is also expressed at the plasma membrane^26–31^.

Furthermore, we demonstrated that the peptide-binding domain of GRP78 was the region that interacted with MC4R. Given that this domain contributes to the chaperone functions of GRP78 through its interactions with target proteins, GRP78 may contribute to the modification or trafficking of MC4R and thus regulate the changes in MC4R signaling. Indeed, when cells were treated with siGRP78, both MC4R trafficking and MC4R-induced cAMP-mediated signaling were altered. These data illustrate the importance of fine-tuning the expression level of GRP78, which in turn can modulate the trafficking or intracellular signaling of MC4R. Furthermore, in cells under ER stress, which have induced GRP78 protein expression, MC4R trafficking by its agonist was severely disturbed. MC4R trafficking was further disrupted when ER stress induction was combined with GRP78 knockdown. If ER stress is too severe or chronic, the ability of the UPR to properly manage protein folding homeostasis may be compromised, and the induction of GRP78 under this ER situation may result in an alteration of MC4R trafficking and signaling. The fact that the interaction between MC4R and GRP78 was reduced under ER stress induction in vivo (Fig. 4) supports this hypothesis.

The delicate regulation of GRP78 expression levels has probably contributed to the contradictory observations made by several studies that examined the physiological relevance of GRP78. For example, Ye et al. reported that *Grp78* heterozygosity increased energy expenditure and attenuated HFD-induced obesity^32^. *Grp78*^+/−^ mice were resistant to diet-induced hyperinsulinemia, liver steatosis, white adipose tissue (WAT) inflammation, and hyperglycemia. The authors thus explained that *Grp78* heterozygosity in WAT under HFD stress promoted an adaptive UPR, attenuated translational block, and upregulated ER degradation-enhancing α-mannosidase-like protein and ER chaperone expression, all of which improved ER quality control and folding capacity^32^. In contrast, Contreras et al. demonstrated that genetic overexpression of GRP78 in the rat hypothalamus abolished ceramide action by reducing hypothalamic ER stress and increasing brown adipose tissue thermogenesis, leading to weight loss and improved glucose homeostasis^33^. Furthermore, overexpression of GRP78 in the hypothalamus of obese Zucker rats resulted in reduced body weight by increasing BAT thermogenesis, decreasing leptin and insulin resistance, and reducing hepatic steatosis^33^. Therefore, a rather contradictory role has been observed for GRP78 in the hypothalamus. Additional recent reports by Contreras et al. indicated that genetic overexpression of GRP78, specifically in the ventromedial nucleus of the hypothalamus, was sufficient to alleviate ER stress and to reverse the obese and metabolic phenotype. The effects were independent of feeding and leptin signaling, but were related to increased thermogenic activation of brown adipose tissue and induction of browning in WAT^34^. In the present study, knockdown of GRP78 in the PVN did not affect ND-fed animals. However, the body weight of HFD-fed mice was significantly higher than that of the control group. Interestingly, this increase in body weight did not accompany a change in food intake or oxygen consumption, raising the possibility that GRP78-mediated fat browning is involved as previously suggested^34^.

Interestingly, although GRP78 is a molecular chaperone and is known to be widely expressed, there is increasing evidence showing its specific expression in the hypothalamus in relation to proteins involved not only in energy homeostasis, but also in other homeostatic regulation processes. The proprotein convertase 1/3 (PC1/3) mutant (N222D) has recently been reported to bind to GRP78, and GRP78-mediated PC1/3-N222D degradation is induced in pancreatic islets, the pituitary, and the hypothalamus, which contributes to the obese phenotype observed in a mouse model harboring the hypomorphic mutation N222D^35^. Additionally, GRP78 mRNA is reportedly expressed in neurons in the hypothalamic PVN that produce arginine vasopressin (AVP), an antidiuretic hormone, which is also involved in energy homeostasis^36^. Interestingly, PVN AVP neurons are known to interact with the melanocortin system to regulate feeding behavior^37^, and more recently, the colocalization of AVP and GRP78 has been associated with the regulation of ER-associated degradation of AVP in the hypothalamus^38^. These lines of evidence led to the hypothesis that the ability of GRP78 to target specific proteins in specific neuronal populations is responsible for protein trafficking and/or clearing in the brain and may be associated with diverse physiological processes.

Chemical chaperones have been shown to increase ER function and reduce ER stress^39^, and chemical chaperones such as 4-PBA can relieve ER stress in liver and adipose tissues and enhance insulin sensitivity in a mouse model of severe obesity and type 2 diabetes^40^. Chemical chaperones have also been shown to act as leptin-sensitizing agents by improving central leptin resistance in diet-induced obese mice^19,41^. In an attempt to regulate MC4R for therapeutic purposes, pharmacological chaperones (other than 4-PBA) have been tested. In receptor-transfected cell culture systems, these chaperones have been shown to rescue the trafficking and signaling of mostly mutant MC4Rs^42–45^. Given that GRP78 appeared to be able to regulate MC4R function in vivo, in association with the role of GRP78 as a molecular chaperone, we assessed the effect of a chemical chaperone, 4-PBA, on the MC4R-mediated control of body weight and food intake. The duration of 4-PBA treatment in animal varies in the literature from several days to several weeks with an intraperitoneal injection of 100–200 mg/kg/day^46–48^. Here, we optimized the treatment dose and performed our study using 5 days of 200 mg/kg/day 4-PBA delivered with an i.p. injection, followed by the analysis of the MC4R-mediated control of body weight and food intake with administration of an MC4R agonist or antagonist. Indeed, we observed that 4-PBA pretreatment overcame the antagonistic effects of AgRP and promoted and prolonged the effects of the MC4R agonist to control food intake and body weight. These data, in parallel with the known chaperone function of GRP78, further support the therapeutic targeting of chaperones in the treatment of metabolic disorders and identify GRP78 as a cellular modulator of MC4R in the control of energy homeostasis.

## Electronic supplementary material

Supplementary Materials and Methods
