# Supplementary material for: Glucose-regulated protein 78 binds to and regulates the melanocortin-4 receptor
Source: Exp Mol Med. 2018 Sep 12;50(9):120. doi: 10.1038/s12276-018-0144-8 (PMC6135830; doi:10.1038/s12276-018-0144-8)
Supplement: Supplementary file 1 — Supplementary Materials and Methods [file 12276_2018_144_MOESM1_ESM.docx]

# Supplementary Materials and Methods

*Cell culture and ER stress induction*

HEK 293T cells were grown in Dulbecco’s Modified Eagle Medium (DMEM) (WelGENE, Inc.), supplemented with 10% fetal bovine serum (WelGENE Inc.) and penicillin-streptomycin solution (100 µg/ml streptomycin, 100 U/ml penicillin G) (WelGENE Inc.), at 37°C with 5% CO_2_. To induce ER stress, cells were treated with 2.5 µg/ml of tunicamycin for three hours in the medium, supplemented with 0.1% bovine serum albumin and 0.1% antibiotics to serum-starve the cells. All cell works were performed in status of minimizing contamination: using autoclaved instruments and medium containing antibiotics.

To detect GRP78 (1:1000, Santa Cruz, sc-1050 or 1:5000, Cell Signaling, 3177), X-box binding protein-1s (XBP-1s) (1:1000, Cell Signaling, 12782), phosphorylated inositol requiring enzyme 1α (pIRE1α) (1:1000, Novus biologicals, NB100-2323), and phosphorylated eukaryotic initiation factor 2α (peIF2α) (1:1000, Cell Signaling, 3398) expression in cell lysates, 30 μg of lysates were loaded for each sample.

*Mice*

Ten–twelve-week-old male C57BL/6J *ob/ob* mice and wild-type (WT) mice (Jackson Laboratories, Bar Harbor, ME) were adapted to the environment for 1 week prior to the study. All animals were housed under a 12-h light/dark cycle under constant conditions of temperature and humidity, and had access to tap water (membrane filtered purified and autoclaved water) and regular diet (Purina Certified Rodent Diet, USA) *ad libitum*. For diet-induced obese (DIO) mice experiment, 10-week-old male C57BL/6J mice mice were randomly divided into a control group (fed normal diet (ND)) and a HFD group (fed a high-fat diet (HFD) (58.0% fat; D12331; Research Diets) After 13 weeks of HFD feeding, the mice in the HFD group with obesity (the body weight is high over 20% of the mean value of the control mice’ body weight, with average body weight of 36.07+1.30g (n= 5) as compared to ND group with 26.40+0.49g (n= 4). For the induction of ER stress, similarly, 10–14-week-old male C57BL/6J mice were fed HFD for 20 weeks, after 20 weeks of HFD, the body weight of the mice in the HFD group with obesity (40.08+0.10g, n=31) was significantly higher than that of ND group (28.48+0.08g, n=31). Body weight and food intake were measured every day at 5:00 p.m. Prior to sacrifice, 24 h of oxygen consumption (Panlab, Oxylet System) and 1 h of locomotor activity (Med Associates) were measured. Mouse procedures were performed in accordance with standards approved by the Institutional Animal Care and Use Committee of Korea University.

*Plasmid construction for GRP78 domain*

Sequence of the Chinese hamster GRP78 cDNA was obtained from the NCBI database (M17169). GlobPlot algorithm was used to design the following GRP78 functional domains: 1st domain (D1) a.a 1–252, 2nd domain (D2) a.a 253–385, 3rd domain (D3) a.a 386–502, and 4th domain (D4) a.a 503–654. Plasmids for each domain fragment were constructed by PCR; each fragment was amplified using the Chinese hamster GRP78 DNA as the template and with the forward and reverse primers shown in Supplementary Table 1. The PCR products were inserted between the BamHI and XhoI restriction sites of the pET28-a expression vector (Novagen).

*Knockdown of GRP78*

To knockdown GRP78 gene expression, HEK 293T cells were grown to 50–60% confluence in a 100 mm dish and transfected with 6 µg of siRNA against GRP78 (siGRP78, pSilencer 2.1-U6 vector) and 1 µg of pCH110 plasmid carrying the β-galactosidase gene using PEI, or cells were transfected with 1ug of siGRP78 and 0.5µg of pCH110 in a 6 well plate using Lipofectamine3000 (Invitrogen). Four hours after transfection, the media was replaced with a fresh growth medium and incubated for 48h–72 h. The target site of the siRNA against GRP78 was identified by the siRNA finder (Ambion). The siRNA sequence for GRP78 was 5′- AATCAAGGTCTATGAAGGTGA-3′; this sequence was also used to design the lentiviral vector encoding a short hairpin RNA specific to the GRP78 mRNA (Lenti-shGRP78).

*Preparation and titration of lentiviral vectors*

Lentiviruses were prepared by the transient transfection of three plasmids (pLL3.7, VSVG, and **△**8.9) in HEK 293T cells. Media containing the virus was harvested at 24 h, 48 h, and 72 h after transfection, and concentrated using 10% polyethylene glycol and pelleted by ultracentrifugation (29,000 rpm, 2 h, 4℃). The lentivirus titers were determined by fluorescence-activated cell sorting analysis.

*Bacterial protein expression and purification*

DNA encoding the third intracellular loop of MC4R gene was cloned into the pGEX vector (GE Healthcare Life Sciences) for GST-tagged protein (GST-MC4Ri3) expression. The various globular domain fragments of GRP78 were cloned into pET28a expression vector to produce NH2-terminal 6X His-tagged fusion proteins, consisting of six histidine residues (His-GRP78). GST tag alone, GST-MC4Ri3, and His-GRP78 fragments were transformed into BL21 (DE3) bacterial cells. Small-scale cultures were grown overnight and used to inoculate a fresh Luria-Bertani medium. The new cultures were subsequently grown for 1.5 h, induced with 1 mM isopropyl-1-thio-β-D-galactopyranoside, and grown for an additional 4 to 6 h. Cells were harvested by centrifugation and resuspended in 3% of the original volume in either phosphate-buffered saline (PBS) containing protease and phosphatase inhibitors (1 µg/ml leupeptin, 1 µg/ml aprotinin, 1 mM Na3VO4, and 1 mM PMSF) for GST-fusion proteins or in Tris-HCl (pH 7.5) containing protease and phosphatase inhibitors for His-tagged protein. Next, cells were extracted by sonication and incubated with 1% Triton X-100 to disrupt the cell membrane. The lysates were clarified by centrifugation, and GST-fusion and His-tagged proteins were purified on Glutathione Agarose 4B columns (Peptron, Inc.) and nickle-nitrilotriacetic acid (Ni-NTA) agarose columns (Qiagen), respectively.

*GST- and His-tag pull-down assays*

For GST-tag pull-down assays, GST-fusion proteins were immobilized for 2 h onto GST-agarose beads. The beads were then washed 3 times with PBS (pH 7.4) containing 1% Triton X-100 and protease and phosphatase inhibitors. Subsequently, purified His-tagged proteins were added and incubated overnight. Unbound proteins were washed 3 times, and the bound proteins were eluted and diluted in the protein sample buffer (2% SDS, 5% β-mercaptoethanol) for analysis by SDS-PAGE. The precipitation obtained from GST-pull down with hypothalamic proteins was developed by silver staining. Specific 70 kDa band for the third intracellular loop of MC4R was cut out by in-gel trypsin digestion and subjected to proteomic analysis, matrix assisted laser desorption/ionization time-of-flight mass spectrometry (MALDI-TOFMS).

HEK 293T cells transiently transfected with 3xFlag-MC4R plasmids (MC4R cloned into p3xflag-cmv10 vector) were lysed in cell lysis buffer (50 mM Tris-HCl [pH 7.5], 150 mM NaCl, 2 mM EDTA, 2.5 mM sodium pyrophosphate, 0.5% sodium deoxycholate, 1% Triton X-100, 1 mM β-glycerophosphate, and protease and phosphatase inhibitors) and centrifuged to remove cellular debris. For His-tag pull-down assays, His-tagged proteins were immobilized for 2 h with Ni-NTA agarose, and then incubated overnight with cell lysates containing the 3xFlag-MC4R proteins. Unbound proteins were removed by washing 3 times with 20 mM Tris-HCl (pH 8.0) buffer containing 200 mM NaCl and 1% Triton X-100. Bound proteins were eluted into protein sample buffer for analysis by SDS-PAGE.

*Membrane fractions of HEK 293T cells and hypothalamic lysate preparation*

HEK 293T cells were grown in 100 mm dishes until 70% confluence was reached. The cells were transiently co-transfected with 6 µg of 3xFlag-MC4R and 4 µg of GRP78-Myc plasmids (GRP78 cloned into pcDNA6-mychisA vector). Thirty-six hours after transfection, the cells were washed with ice-cold PBS, and then harvested by centrifugation at 1000 × g for 5 min. The supernatant was discarded and the cell pellet was resuspended in Tris-EDTA buffer (10 mM Tris-HCl [pH 7.5] and 5 mM EDTA). The cells were homogenized with a Polytron homogenizer (IKA) and the homogenates were centrifuged at 1000 × g for 10 min. The supernatants were collected and centrifuged at 45000 rpm for 40 min to separate the plasma membrane fraction. The pellets were washed once with Tris-EDTA solution and centrifuged again at 45000 rpm for 40 min. The final pellet containing the membrane proteins was resuspended in 50 mM Tris-HCl (pH 7.7).

After administration of ER stressors such as HFD (for 20 weeks) or tunicamycin (intracerebroventricular (icv) injection, 80ug x 2times every 6hr), mice were sacrificed and their brains were removed. Hypothalami were dissected and homogenized in lysis buffer (20 mM Tris [pH 7.5], 200 mM NaCl, 1 mM EDTA, 1% Nonidet P-40, 1% Triton X-100, and protease and phosphatase inhibitors) using a dounce hand homogenizer. Hypothalamic homogenates extracted from 3–4 mice were centrifuged for 30 min at 4°C at 12000 rpm and supernatants were placed in a fresh tube.

*ELISA for quantification of Flag-tagged MC4R on the cell surface*

3xFlag-MC4R on the cell surface was quantified by ELISA to measure MC4R internalization. HEK 293T cells were seeded in Poly-(d)-Lysine-coated 12-well plates with culture medium and transiently transfected with 3xFlag-MC4R plasmid using PEI. After 36 h of transfection, cells were incubated in DMEM with mouse anti-Flag antibody (1:2500) for 2 h at room temperature to label cell surface receptors. The cells were then washed with DMEM to remove unbound antibody, and incubated at 37°C with 1 µM Acetyl-(Nle4, Asp5, D-Phe7, Lys10)-cyclo-α-MSH (4-10) amide acetate (Melanotan II; MTII) for 0, 5, 10, 20, or 40 min. Incubations were terminated by placing the plates in an ice bath. The cells were then washed twice with ice-cold PBS and fixed for 10 min at room temperature with 3% paraformaldehyde in PBS, pH 7.4. Nonspecific binding sites were blocked for 30 min at room temperature with PBS containing 1% bovine serum albumin. To measure the total and cell surface 3xFlag-MC4R, cells were blocked in PBS containing 1% bovine serum albumin, with 0.2%Triton X-100 (permeabilized) or without (unpermeabilized), respectively, for 30 min at room temperature.^1^ After blocking, the cells were incubated for 60 min at room temperature with goat anti-mouse IgG horseradish peroxidase conjugate (1:2500) in blocking buffer with 0.2%Triton X-100 (permeabilized) or without (unpermeabilized). After three additional washings with PBS, MC4R expression was quantified by adding 0.4 ml of horseradish peroxidase substrate (BM Blue, 3,3ʹ-5,5ʹ-Tetramethylbenzidine). OD 450 was measured after a 10 min incubation (against the reference wavelength, 690 nm) at room temperature.

*Intracerebroventricular cannulation and intraperitoneal injection*

For surgery, 10–12-week-old male mice were anesthetized with an intraperitoneal injection of Ketamine (1.6 μl/g) and Rompun (0.05 μl/g) (Bayer) and placed in a stereotaxic apparatus (Kopf Instruments). A 26-gauge guide cannula was implanted using the following coordinates: 0.3 mm posterior and 1 mm lateral relative to the bregma, at a depth of 3.0 mm. Injection position was verified at the end of the experiment via dye administration and histological analysis. After 7 days of recovery, mice were treated 2 times, 6 h apart, with DMSO as control or with 80 µg of tunicamycin in the lateral ventricle. For MC4R agonist or antagonist administration, mice were injected with 3 nmol of MTII (Bachem) or 2 nmol of AgRP (Phoenix pharmaceuticals, Inc.). A chemical chaperone, 4-PBA (Calbiochem), was given by intraperitoneal (200 mg/kg) injection for once a day for 5 consecutive days.

*Microinjection of lentiviral vectors*

Six-week-old male mice were bilaterally or unilaterally microinjected with Lenti-control or Lenti-shGRP78 into the PVN. PVN microinjection was performed using the following coordinates: -0.3 mm posterior, ±0.2 mm lateral, and -5.25 mm ventral from bregma. Approximately 5.0 × 10^5^ virus particles resuspended in 2.0–3.0 μl volume were administered to each side. After surgery, the mice were allowed to recover for 7 days. The data obtained from the mice injected with lentivirus either unilaterally or bilaterally were analyzed.

*Reverse transcription and real-time PCR analysis*

Brain tissue was obtained from the PVN of mice injected with Lenti-control or Lenti-shGRP78. Total RNA was extracted by Trizol and treated with RQ1 RNase-free DNase (Invitrogen). Reverse transcription of RNA was performed using Thermo Scientific Maxima reverse transcriptase. The resulting cDNA was subjected to real-time PCR analysis by using a LightCycler 480 system (Roche, Mannheim, Germany). β-Actin was used to normalize the expression levels of GRP78 mRNA. The primer sequences for GRP78 mRNA were (F) 5′-ATTGGAGGTGGGCAAACCAAG-3′ and (R) 5′-TTGCTTGTCGTCGCTGGGCATCATT-3′. The primers for β-Actin were (F) 5′-ACTATTGGCAACGAGCGGTT-3′ and (R) 5′-TGTCAGCAATGCCTGGGTACAT-3′. All real-time RT-PCR reactions were performed in triplicates and quantitation was expressed as ΔΔCt.^2^

*Stereological cell counts*

The number of GRP78-positive cells was counted using a stereological system (MBF Stereo Investigator, MBF Bioscience, VT, USA). This system provides specialized techniques for extracting quantitative information of a three-dimensional material using measurements made on two-dimensional planar sections of the material or tissue. The sections used for counting covered the entire PVN, from the rostral tip to the caudal end of that region; the PVN was defined as Anterior–Posterior axis, -0.58 to -1.06 mm from the bregma, according to the mouse brain atlas of Paxinos and Franklin, 2001. The total number of neurons was calculated on the basis of the optical fractionator formula.^3^ The quantification data is shown as the average of the total number of neurons/nucleus/animal. Four or five mice of each group were used in experiments with obese mice model.

*Co-Immunoprecipitation*

HEK 293T cells were transiently co-transfected with 3xFlag-MC4R and GRP78-Myc plasmids. Cells were harvested and membrane proteins were prepared as described above. For immunoprecipitation experiments, cell lysates were pre-cleared with Protein G Sepharose beads (GE healthcare Inc.) for 2 h at 4°C. The pre-cleared extracts were incubated with 2 µg of anti-Myc monoclonal antibody (9B11, Cell Signaling) or 5 µg of anti-Flag monoclonal antibody (M2, Sigma) overnight at 4°C. Immunoprecipitations were performed in 20 µl of 50% slurry of Protein G Sepharose beads for 5 h. Antibody-antigen immunocomplexes bound to the Sepharose beads were washed 5 times for 10 min each with washing buffer containing 50 mM Tris-HCl (pH 7.5), 150 mM NaCl, 2 mM EDTA, 1% Triton X-10, and protease and phosphatase inhibitors. Proteins were then eluted into protein sample buffer and separated by SDS-PAGE for analysis.

For the analysis of hypothalamic lysates, 2 mg of each lysate was pre-cleared by Protein G-Sepharose beads (GE healthcare Inc.) and incubated with 10 µg anti-GRP78 polyclonal antibody. The immunoprecipitation was performed as described above. To detect GRP78 (1:500, Santa Cruz, sc-1050), XBP-1 (1:500, Santa Cruz, sc-7160), pIRE1α(1:1000, Novus biologicals, NB100-2323), and peIF2α(1:1000, Cell Signaling, 3398) expression in hypothalamic extracts, 20 μg or 50ug of lysates were loaded for each sample. One hundred micrograms of lysates were used for the detection of MC4R (1:250, Abcam, ab24233 or Alomone labs, AMR-024) expression.

*CRE-luciferase reporter gene assay*

HEK 293T cells grown to 60% confluency in 100 mm dishes were co-transfected with 5 µg of hMC4R plasmid, 5 µg of pCRE-Luc plasmid (Promega), and 1 µg of pCH110 plasmid carrying the β-galactosidase gene, using PEI. Four hours after transfection, the transfection mixture was replaced with a fresh growth medium. Twelve hours after transfection, the cells were seeded in a 12-well plate at a density of 3 × 10^5^ cells/well. Thirty-six hours after transfection, the medium was replaced with serum-free medium supplemented with 0.1% bovine serum albumin and antibiotics to serum-starve the cells. After an overnight incubation, the cells were treated with 2.5 µg/ml of tunicamycin for 3 h, and then with various concentrations of MTII for an additional 3 h. After treatment, the cells were lysed and assayed for luciferase activity using the luciferase assay system (Promega), and luminescence was measured using a 96-well Luminoskan Ascent (Thermo Labsystems). The expression of the reporter gene was normalized using β-galactosidase activity. Results were expressed as the ratio of luciferase activity of the transfected cells to that of the non-stimulated controls. The mean values of the data obtained were fitted to a sigmoid curve with a variable slope factor using nonlinear square regression in GraphPad Prism (GraphPad Software, San Diego, CA).

*Immunofluorescence and confocal microscopy*

For the MC4R and GRP78 co-localization assay, HEK 293 cells expressing 3xFlag-MC4R and GRP78-Myc were seeded on coverslips. After treatment with MTII for the times indicated, the cells were fixed with 4% paraformaldehyde in PBS for 15 min. The cells were rinsed with PBS and blocked for nonspecific binding sites for 30 min at room temperature with PBS containing 2% bovine serum and 0.2% Triton X-100. The cells were then incubated overnight at 4°C with anti-Flag M2 mouse monoclonal IgG and anti-Myc rabbit polyclonal IgG (Cell Signaling Technology) diluted in blocking buffer. The following day, cells were washed with PBS and incubated for 1 h at room temperature with donkey anti-mouse IgG Alexa Fluor 568 (Invitrogen) and donkey anti-rabbit IgG Alexa Fluor 488 (Invitrogen). The cells were also counterstained with Hoechst 33342 for 5 min to label nuclei. After rinsing with PBS containing 0.2% Triton X-100, the coverslips were mounted for fluorescent confocal microscopy. Confocal microscopy was performed on a Zeiss LSM 510 META laser scanning microscope (Carl Zeiss).

*Immunohistochemistry*

For immunohistochemical staining, mice were transcardially perfused with 0.1M PBS (pH 7.4) and then with 4% paraformaldehyde. The brains were removed and post-fixed with 4% paraformaldehyde and cryoprotected in 30% sucrose for 24–48 h. Free-floating cryostat sections of 40 μm thickness were serially prepared for GRP78 and MC4R immunohistochemistry. Sections were washed in 0.1 M PBS (pH 7.4) three times for 10 min and then incubated in 0.1 M PBS solution containing 0.3% H_2_O_2_ and 50% methanol for 30 min. Sections were incubated in blocking solution (0.3% Triton X-100, 1% BSA in 0.1 M PBS) for 30 min, followed by an overnight incubation at 4℃ with the following primary antibodies: goat polyclonal anti-GRP78 (1:500, catalog no. sc-1050, Santa Cruz).Sections were incubated at room temperature in biotinylated secondary antibody (anti-goat IgG, 1:500 or anti-rabbit IgG, 1:500, Vector Laboratories, Burlingame, CA) for 30 min, and then in the ABC kit (Vector Laboratories) for 30 min. Subsequently, the final reaction was developed in DAB (Vector Laboratories). Washing with 0.1 M PBS (3–5 times for 10 min) was performed between each step. Sections were then mounted, dehydrated, and cover-slipped.

*Statistical analysis*

Data are presented as means ± SEM. Two-sample comparison was analyzed using the Student’s *t* test (two- or one-tailed), and multiple comparisons were performed using two-way ANOVA or two-way repeated-measures ANOVA (RM-ANOVA) analysis followed by Bonferroni post hoc test. A P-value of <0.05 was considered statistically significant.

**References**

1 Mohammad S, Baldini G, Granell S, Narducci P, Martelli AM, Baldini G. Constitutive traffic of melanocortin-4 receptor in Neuro2A cells and immortalized hypothalamic neurons. *J Biol Chem* 2007; **282**: 4963-4974.

2 Schefe JH, Lehmann KE, Buschmann IR, Unger T, Funke-Kaiser H. Quantitative real-time RT-PCR data analysis: current concepts and the novel "gene expression's CT difference" formula. *J Mol Med (Berl)* 2006; **84**: 901-910.

3 West MJ. New stereological methods for counting neurons. *Neurobiol Aging* 1993; **14**: 275-285.

**Supplementary Table 1. Oligonucleotide primers used to construct the different GRP78 domain fragments.**

| **Construct** | **Primer** | |
| --- | --- | --- |
| **GRP78 1GD** | (F) | 5ʹ- TA**GGATCC**ATGAAGTTCCCTATGG-3’ |
|  | (R) | 5ʹ-ATA**CTCGAG**TTAATGAGTGTCTCCA-3’ |
| **GRP78 23GD** | (F) | 5ʹ-ATA**GGATCC**CTCGGTGGGGAAGACT-3’ |
|  | (R) | 5ʹ-ATA**CTCGAG**TTACTCAAAGGTGACT-3 |
| **GRP78 12GD** | (F) | 5ʹ-ATA**GGATCC**ATGAAGTTCCCTATGG-3 |
|  | (R) | 5ʹ-ATA**CTCGAG**AGGATGGCTCCTTGCC-3’ |
| **GRP78 3GD** | (F) | 5ʹ-ACA**GGATCC**CGTGGCATAAACCCA-3’ |
|  | (R) | 5ʹ-ATA**CTCGAG**TTACTCAAAGGTGAC-3’ |
| **GRP78 4GD** | (F) | 5ʹ-ATA**GGATCC**ATAGATGTTAATGGTA-3’ |
|  | (R) | 5ʹ-ATA**CTCGAG**TTACAACTCATCTTTT-3 |

Each protein was constructed by PCR with the indicated primers.

The restriction enzyme sites are underlined.

**Supplementary Table 2. Statistical analysis for effect of the chemical chaperone, 4-PBA, on MC4R-mediated regulation of body weight and food intake.**

| (At 10hr) | Body weight (g) | | Food intake (g) | |
| --- | --- | --- | --- | --- |
|  | 4-PBA  -non-treated | 4-PBA-treated | 4-PBA  -non-treated | 4-PBA-treated |
| Vehicle vs. MTII | 2.69 ± 0.31g  vs. 1.75 ± 0.24 g | 3.10 ± 0.32g  vs. 1.64 ± 0.21g | 3.15 ± 0.15 g  vs. 2.45 ± 0.14g | 3.38 ± 0.17g  vs. 2.32 ± 0.16g |
| Vehicle  vs. MTII + AgRP | 2.69 ± 0.31g  vs. 2.91 ± 0.21g | 3.10 ± 0.32g  vs. 1.91 ± 0.27g | 3.15 ± 0.15 g  vs. 3.18 ± 0.14g | 3.38 ± 0.17g  vs. 2.41 ± 0.17g |
| 4-PBS x MTII or AgRP interaction | F_2,48_ = 3.62, P = 0.0344 | | F_2,48_ = 5.26, P = 0.0086 | |

(n = 9, two-way ANOVA, Bonferroni post hoc tests)
